# The importance of rapport and relationship building when recruiting to clinical trials: a qualitative investigation of trial recruitment consultations in a surgical RCT

**DOI:** 10.1186/1745-6215-16-S2-O37

**Published:** 2015-11-16

**Authors:** Lynda Constable, Danielle Pirie, Katie Gillies, Sharon McCann, Suzanne Breeman, Cathryn Glazener

**Affiliations:** 1 University of Aberdeen, Aberdeen, UK

## Background

Pelvic organ prolapse affects the quality of life of a large number of women, yet there is not enough evidence to identify which procedures are best for treating vault or uterine prolapse.

## Aim

The primary aim of this study (VUE-Qual) was to improve understanding of the issues impacting decision making for women invited to take part in a surgical prolapse trial (VUE).

## Methods

Qualitative analysis of audio-recorded recruitment consultations within VUE between potential participants and recruiter. These interactions (n=6) were systematically evaluated using the Framework approach, and the main themes impacting on the decision making process for trial participation were categorised.

## Results

The key findings highlighted the importance of; a) the context to the recruitment consultation, b) the current health status of potential participants at the time of trial invitation, and c) the trial information exchange process. These findings were underpinned by an overarching theme relating to recruiter rapport and relationship building with potential participants. The recruiter demonstrated an important role in terms of being empathetic, reassuring, supportive and attentive when discussing the trial with the participants.

## Conclusions

Previous studies have shown that exploring treatment preferences, within the context of recruitment consultations, facilitated recruitment. VUE-Qual has provided a rich insight into how information is discussed in recruitment consultations between potential participants and recruiter in the context of a surgical prolapse trial. It has also identified aspects of the recruitment consultation that should be explored more systematically in other trial recruitment settings to potentially improve the recruitment process.

